# NATpipe: an integrative pipeline for systematical discovery of natural antisense transcripts (NATs) and phase-distributed nat-siRNAs from *de novo* assembled transcriptomes

**DOI:** 10.1038/srep21666

**Published:** 2016-02-09

**Authors:** Dongliang Yu, Yijun Meng, Ziwei Zuo, Jie Xue, Huizhong Wang

**Affiliations:** 1 College of Life and Environmental Sciences, Hangzhou Normal University, Hangzhou 310036, PR China; 2 Zhejiang Provincial Key Laboratory for Genetic Improvement and Quality Control of Medicinal Plants, Hangzhou Normal University, Hangzhou 310036, China

## Abstract

Nat-siRNAs (small interfering RNAs originated from natural antisense transcripts) are a class of functional small RNA (sRNA) species discovered in both plants and animals. These siRNAs are highly enriched within the annealed regions of the NAT (natural antisense transcript) pairs. To date, great research efforts have been taken for systematical identification of the NATs in various organisms. However, developing a freely available and easy-to-use program for NAT prediction is strongly demanded by researchers. Here, we proposed an integrative pipeline named NATpipe for systematical discovery of NATs from *de novo* assembled transcriptomes. By utilizing sRNA sequencing data, the pipeline also allowed users to search for phase-distributed nat-siRNAs within the perfectly annealed regions of the NAT pairs. Additionally, more reliable nat-siRNA loci could be identified based on degradome sequencing data. A case study on the non-model plant *Dendrobium officinale* was performed to illustrate the utility of NATpipe. Finally, we hope that NATpipe would be a useful tool for NAT prediction, nat-siRNA discovery, and related functional studies. NATpipe is available at www.bioinfolab.cn/NATpipe/NATpipe.zip.

## Brief introduction of NATs (natural antisense transcripts) and nat-siRNAs (small interfering RNAs originated from NATs)

Natural antisense transcripts (NATs) are pairs of complementary transcripts encoded by the endogenous genes of plants or animals. The NATs could be protein-coding or non-protein-coding (non-coding) transcripts. Relying on the high complementarity of the annealed regions, the NAT pairs are thermodynamically stable *in vivo*. For the organisms with annotated reference genomes, the NATs could be classified into *cis*- or *trans*-NATs according to their genomic origins. A pair of *cis*-NATs is formed by two transcripts derived from two overlapping genomic loci on the opposite strands, while a *trans*-NAT pair is constituted by the complementary transcripts encoded by two distant genomic loci. Thus, one of the distinguishable features between *cis*- and *trans*-NAT pairs is that the complementary regions of the *cis*-NAT pairs are perfectly annealed while the *trans*-NAT pairs usually have mismatches within their annealed regions. However, one common feature is shared by the two NAT categories that the annealed regions should be long and stable enough to ensure the correct formation of the transcript pairs. To date, many organisms do not have their reference genomes, resulting in a big obstacle for NAT identification and classification.

Ten years ago, Zhu’s lab reported that through 24- and 21-nt nat-siRNA-mediated target cleavages, a *cis*-NAT pair could modulate salt tolerance in *Arabidopsis* (*Arabidopsis thaliana*)^1^. During the past few years, growing evidences pointed to the functional involvement of NATs and nat-siRNAs in diverse biological processes. In rice (*Oryza sativa*), Jabnoune and his colleagues identified a *cis*-NAT pair constituted by *PHOSPHATE1;2* (*PHO1;2*) and *cis*-NAT*PHO1;2*. The intriguing finding is that *cis*-NAT*PHO1;2* has an unexpected role in promoting the translation of its complementary partner *PHO1;2*, which could affect the phosphate homeostasis of rice^2^. Systematical study on long non-coding natural antisense transcripts (lncNATs) in *Arabidopsis* uncovered a total of 37,238 NAT pairs. Hundreds out of these NAT pairs exhibited light-responsive expression patterns which were potentially resulted from histone acetylation on the NAT gene loci^3^. In animals, NATs have been proposed to be implicated in alternative splicing of pre-mRNAs, microRNA (miRNA) binding site mimicking, chromatin remodeling, and double-stranded RNA-dependent processes such as RNA editing and RNA interference^4^,^5^,^6^,^7^.

In view of the biological significance of the NATs, several research groups have made great efforts to set feasible criteria for computational identification of NAT pairs from genome or transcriptome sequencing data^3^,^8^,^9^,^10^, and the NAT databases have been established for both animals and plants^11^,^12^. AntiHunter 2.0 is a bioinformatics tool for fast and sensitive extraction of antisense transcripts from BLAST outputs^8^. Unfortunately, it was designed for EST (expressed sequence tag) sequencing data, and the maximum query size was limited to 3 MB. Moreover, AntiHunter 2.0 is currently unavailable online (http://bioinfo.crs4.it/AH2.0). For the model plant *Arabidopsis*, both *cis*- and *trans*-NATs have been systematically predicted^3^,^10^. However, although the criteria for NAT prediction are provided in the previous reports, the computational programs are not available for researchers. As mentioned above, many organisms do not possess reference genomes. Thus, developing a program for NAT prediction for the non-model organisms might be more anxious for the scientists not competent for programming. Followed by NAT identification, searching for the nat-siRNAs becomes a major task since many NATs exert biological roles through siRNA-guided target cleavages or chromatin modifications^1^,^4^,^7^,^13^,^14^,^15^,^16^,^17^,^18^,^19^. To our knowledge, no integrative pipeline has been available for both NAT prediction and nat-siRNA identification. Although some of the NAT databases, such as PlantNATsDB^11^, provide graphic view of the small RNA (sRNA) loci within the annealed regions of the NAT pairs, they do not provide users with detailed genomic arrangement of these sRNA loci and further evidences for extracting reliable nat-siRNA candidates.

## An integrative pipeline for NAT and phased nat-siRNA discovery

**Figure 1:**
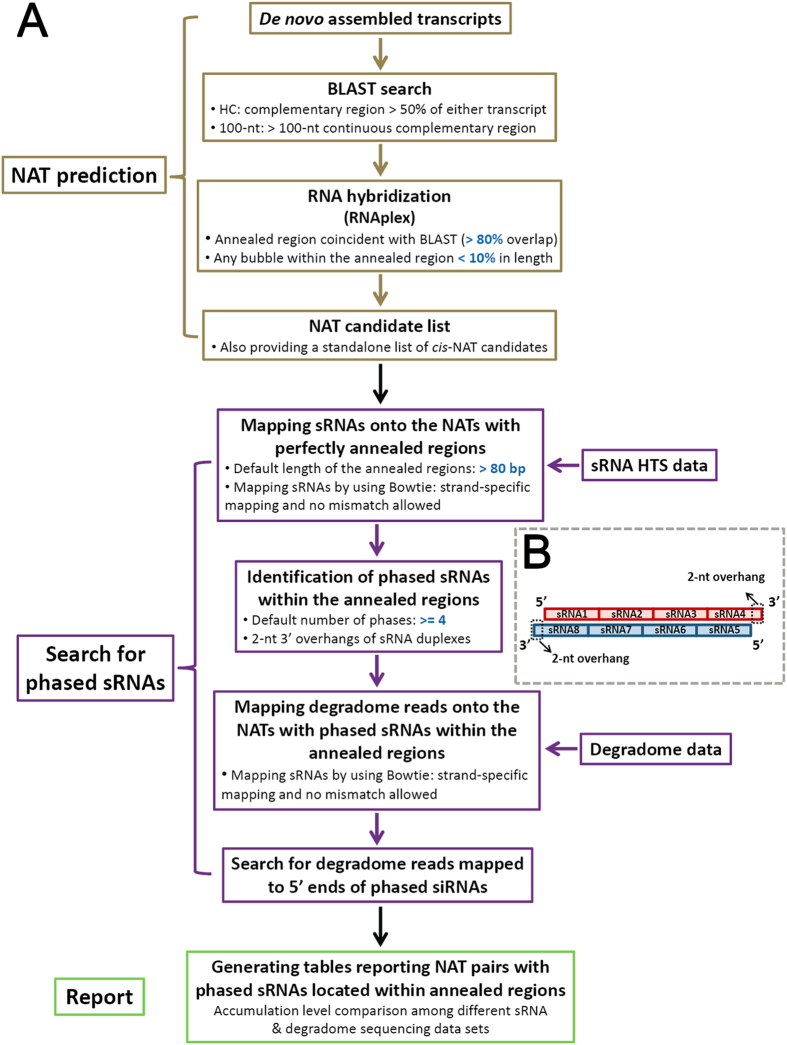
Summarized workflow of NATpipe. (**A**) Two functional modules “NAT prediction” and “Search for phased sRNAs” were integrated into the pipeline. The first module requires *de novo* assembled transcripts as the input, and the second module requires small RNA (sRNA) HTS data (at least) and degradome HTS data (would be best if available). The parameters in blue color are adjustable. (**B**) Illustration of the phase-distributed sRNAs identified within the perfectly annealed regions (>80 bp) of a NAT pair. As an example, eight sRNAs with consistent sequence length were assigned to four phases (four sRNAs on each strand). Each phased sRNA duplex (sRNA1/sRNA8, sRNA2/sRNA7, sRNA3/sRNA6 and sRNA4/sRNA5) possesses 2-nt overhangs at their 3′ ends (dashed boxes just indicate two 2-nt overhangs for example).

In this study, by integrating several existing programs such as BLAST^20^, RNAplex^21^ and Bowtie^22^, we developed a pipeline named NATpipe, allowing users to do a systematical search for the NATs in the organisms without reference genomes. More importantly, following NAT predictions, the pipeline enables users to identify the phase-distributed nat-siRNA loci within the perfectly annealed regions of the NAT pairs based on sRNA and degradome sequencing data (Fig. 1). Considering the lack of the reference genomes, the criteria previously used for *trans*-NAT prediction^11^ were adopted by our pipeline. The prediction starts from BLAST search (see user manual in Data S1 for parameter setting), treating the *de novo* assembled transcripts as the input data. Based on the BLAST results, the predicted NATs will be classified as “HC” (high coverage; the complementary region is longer than 50% of either transcript of the NAT pair) or “100-nt” (the consecutive complementary region of the NAT pair should be 100 nt or longer). Then, RNAplex (see user manual in Data S1 for parameter setting) is employed to verify the annealing potential of the BLAST-predicted NAT pairs at the secondary structure level. After comparison between the BLAST- and the RNAplex-derived results, the NATs fulfilling the two criteria are retained for further analysis: (1) the RNAplex-predicted annealed region of the NAT pair should overlap at 80% or more with the BLAST-predicted complementary region; (2) for the NAT pair predicted by RNAplex, any bubble within the annealed region should be no larger than 10% of this region. As a result, a NAT list along with the detailed information of the hybridized transcripts and their annealed regions will be available for the users. According to the RNAplex prediction, if a NAT pair has a single perfectly annealed region, it will be regarded as a *cis*-NAT pair candidate albeit the lack of the genomic information.

Nat-siRNAs were reported to be implicated in regulating gene expression through target cleavages or chromatin modifications in both animals and plants^1^,^4^,^7^,^13^,^14^,^15^,^16^,^17^,^18^,^19^. On the other hand, the annealed regions of the NAT pairs are the hotspots for the generation of nat-siRNAs with variable sequence length^7^,^16^,^23^,^24^,^25^,^26^,^27^,^28^,^29^,^30^. However, we recognized that it would not make any sense for the biologists just by showing them the hotspots of siRNA loci based on sRNA sequencing data. And, it will be a hard task for us to obtain evidences to support the scattered siRNA loci without any regular distribution patterns on the NATs. The pioneering work by Zhu’s lab attracted our attention that at least a portion of nat-siRNAs was distributed with defined phases^1^. Our previous work on *Arabidopsis* and rice also revealed several pairs of *cis*-NATs encoding phased nat-siRNAs^13^. Consistently, several recent works also unveiled many phase-distributed nat-siRNA loci in plants^16^,^31^. In this consideration, we developed a program to search for phased nat-siRNAs within the perfectly annealed regions of the NATs, which was integrated into NATpipe (Fig. 1B). If the users have sRNA high-throughput sequencing (HTS) data, NATpipe could be continued to identify the phased nat-siRNA loci. First, the NAT pairs with perfectly annealed regions exceeding a predefined length (an adjustable parameter; default: >80 bp) will be extracted. Then, the sRNAs from HTS data sets (see user manual in Data S1 for the required format of the HTS data) will be mapped onto these NATs. Bowtie (see user manual in Data S1 for parameter setting) is employed for the strand-specific mapping, and the perfectly aligned sRNAs will be retained to search for the phased nat-siRNAs. Based on the previous reports, the annealed regions of the NAT pairs are processed by DCL (Dicer-like) proteins^1^,^16^,^17^,^18^,^19^, resulting in the production of phased sRNA duplexes with 2-nt 3′ overhangs. This biogenesis pathway is similar to that of the *trans*-acting siRNAs encoded by the *TAS* genes^32^. In this regard, NATpipe recruited a Perl script to search for phased sRNA duplexes with 2-nt 3′ overhangs within the perfectly annealed regions based on the sRNA mapping result (Fig. 1B). The number of the consecutively distributed sRNA duplexes is another adjustable parameter (default: > = 4). Notably, both the sRNAs displaying spatio-temporal expression patterns and the weakly expressed ones might be rarely detectable due to the limited HTS data utilized for this study. Thus, it might be excessively demanded to set a criterion that both strands of each phased sRNA duplex should be evidenced by sRNA HTS data. Instead, we set a more flexible criterion that sequencing evidences should be obtained for either strand of each phased sRNA duplex, which enabled users to identify much more phased nat-siRNA candidates.

Degradome sequencing (degradome-seq) is a high-throughput strategy for detection of the degraded RNA intermediates. It is worth mentioning that the degradome-seq libraries include poly(A)-tailed remnants generated during DCL-mediated processing of the sRNA precusors^33^,^34^. Thus, we could find evidences from the degradome-seq data to support the processing of the phased nat-siRNA duplexes by DCLs. If users have degradome-seq data (see user manual in Data S1 for the required format of the HTS data), NATpipe could perform strand-specific mapping of the degradome signatures onto the NATs with phased nat-siRNA loci. Only the perfectly matched signatures will be retained. The degradome signatures with their 5′ ends mapped to the 5′ ends of the phased nat-siRNAs, or mapped to the nucleotides next to the 3′ ends of the last phased nat-siRNAs will be regarded as the evidences supporting specific nat-siRNA loci. However, one be noticed that most of the degradome-seq libraries were prepared from poly(A)-tailed RNAs, which could not be utilized for detecting the processing signals from the non-Pol II-transcribed transcripts.

NATpipe, developed by the Perl language, is compatible for both Windows and Linux operation systems. It is freely available at www.bioinfolab.cn/NATpipe/NATpipe.zip.

## NATpipe utility: a case study on the non-model plant *Dendrobium officinale*

To verify the utility of NATpipe for NAT and nat-siRNA discovery, we performed a case study by using RNA sequencing (RNA-seq) data of *Dendrobium officinale*, a non-model plant species. In our recent study, eight RNA-seq data sets [two biological replicates for each of four organs including root, stem, leaf and flower; NCBI SRA (http://www.ncbi.nlm.nih.gov/sra) accession IDs: SRR2014227, SRR2014230, SRR2014236, SRR2014246, SRR2014297, SRR2014325, SRR2014396 and SRR2014476] were generated, providing 445,430,002 valid reads^35^. Although the draft genome of *Dendrobium officinale* has been reported^36^, the genome assembly and the gene annotations are still far from a satisfied status. Thus, we previously took a *de novo* strategy for *Dendrobium* transcriptome assembly by utilizing the eight RNA-seq data sets. As a result, 536,558 transcripts ranging from 201 to 21,555 nt were obtained^35^. In the present study, the 536,558 transcripts were treated as input data for NAT prediction by using NATpipe. As a result, a total of 2,651,469 transcript pairs containing highly complementary regions were identified from the *Dendrobium* transcriptome based on the BLAST results. These complementary pairs were further classified into 1,269,633 “HC” and 1,741,803 “100-nt” pairs. Then, RNAplex was employed for secondary structure prediction to investigate the annealing potential of the BLAST-predicted transcript pairs. The results of RNAplex were parsed and made a comparison with those of BLAST, and the transcript pairs fulfilling the criteria proposed above were retained as the NAT candidates. As a result, a total of 636,074 NAT pairs were retained, and 436 were identified as *cis*-NAT pairs considering the single perfectly annealed region for each pair.

Next, we searched for the phased nat-siRNAs by utilizing eight sRNA HTS data sets (two biological replicates for each of four organs including root, stem, leaf and flower; NCBI SRA accession IDs: SRR2014142, SRR2014143, SRR2014477, SRR2014478, SRR2014146, SRR2014147, SRR2014148 and SRR2014149) reported in our recent study^35^. To do this, the NATs containing perfectly annealed regions longer than 80 bp were subjected to sRNA mapping. A total of 193,167 annealed regions assigned to 163,654 NAT pairs were included in this analysis. Based on the mapping results, NATpipe searched for the sRNA clusters constituted by four or more phased nat-siRNA candidates. As a result, 36,033 phased nat-siRNAs assigned to 8,499 clusters were identified within 1,191 annealed regions of 1,034 NAT pairs.

Then, four degradome-seq data sets (four organs including root, stem, leaf and flower; NCBI SRA accession IDs: SRR2012529, SRR2012531, SRR2012580 and SRR2012592) were used to find evidences supporting the processing of the nat-siRNAs from the NATs. Degradome signatures were mapped onto the NAT pairs encoding phased nat-siRNAs. Based on the mapping results, NATpipe searched for the degradome signatures with their 5′ ends mapped to the 5′ ends of the nat-siRNA candidates, or mapped to the nucleotides next to the 3′ ends of the last phased nat-siRNAs. As a result, 5,739 nat-siRNA candidates (assigned to 3,770 clusters within 524 annealed regions of 502 NAT pairs) were supported by degradome signatures. Finally, a result summary and a detailed report showing the NAT pairs generating phased nat-siRNAs along with degradome-seq evidences were generated.

**Figure 2:**
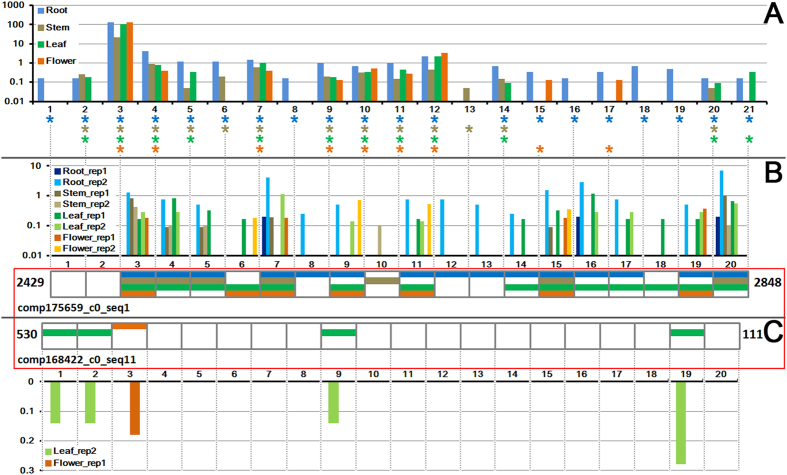
Graphic presentation of the exemplified output results of NATpipe. (**A**) Degradome signatures mapped to the 5′ ends of the phased nat-siRNAs in *Dendrobium officinale* are expressed by asterisks (blue, brown, green and orange for root, stem, leaf and flower respectively). The degradome signal intensity is shown in the histogram. A total of 20 phases were identified within the perfectly annealed region (marked by a red box) between the two transcripts comp175659_c0_seq1 (annealed from 2429^th^ to 2848^th^ nucleotide) and comp168422_c0_seq11 (from 111^th^ to 530^th^ nucleotide) based on small RNA (sRNA) sequencing data. For each phase on a strand of the annealed region, the presence of a nat-siRNA in a specific organ is expressed by a colored bar (blue, brown, green and orange for root, stem, leaf and flower respectively). Based on the sRNA sequencing data, expression levels of the nat-siRNAs are shown in the histograms in **(B**) (for the siRNAs on comp175659_c0_seq1) and (**C**) (for the siRNAs on comp168422_c0_seq11). There are two biological replicates of the sRNA sequencing experiments. Please note, the *y* axes of the three histograms are measured in RPM (reads per million) with exponential increment.

An example of output result reporting a NAT pair producing phased nat-siRNAs is shown in Table S1. It provides us with the following information: (1) Based on the RNAplex-predicted annealed region between comp175659_c0_seq1 (from 1,904^th^ to 2,956^th^ nucleotide) and comp168422_c0_seq11 (from 1^st^ to 1,053^th^ nucleotide), the two transcripts might form a NAT pair. (2) Combinatory use of sRNA-seq data from four organs of *Dendrobium* enabled us to identify 63 nat-siRNAs assigned to 20 consecutive phases. However, when using sRNA-seq data from a single organ, eight nat-siRNAs assigned to seven phases were identified in roots (highlighted in gray background in Table S1), and nine nat-siRNAs assigned to seven phases (gray background) and 12 nat-siRNAs assigned to seven phases (gray background) were identified in leaves. (3) Detailed information of the degradome signatures (including IDs, expression levels and positions on the NATs) supporting the processing of the nat-siRNAs is also provided. Based on Table S1, an intelligible figure could be drawn to display phased nat-siRNAs and degradome signatures with organ-specific patterns (Fig. 2). Intriguingly, 58 out of 63 nat-siRNAs detected from eight sRNA HTS data sets were assigned to comp175659_c0_seq1, forming 18 consecutive phases. Only five nat-siRNAs were assigned to comp168422_c0_seq11. Similar to our result, a previous study on *Arabidopsis* and rice reported that the nat-siRNAs frequently distributed with a strand bias within the overlapping regions of the *cis*-NATs^16^.

## Concluding remarks and perspectives

Here, we provide researchers with an integrative pipeline for NAT prediction by using *de novo* assembled transcriptomes of the non-model plant species. NATpipe also allows users to search for phased nat-siRNAs depending on the availability of sRNA and degradome sequencing data. The publicly available, widely used tools (BLAST, RNAplex and Bowtie), the adjustable parameters, and the detailed user manual ensure the ease of use of NATpipe. NATpipe was written by Perl language, and was compatible for Windows and Linux operation systems. The phased nat-siRNAs along with the degradome-seq evidences facilitate researchers to design further experiments for functional studies on the NATs and the siRNAs.

We acknowledge that the endogenous small interfering RNA (endo-siRNA) system of animal has not been characterized as well as that of plants, and the two systems may be different in some aspects. But, increasing evidences demonstrated that in addition to the endo-siRNA pathway previously identified in the nematodes, intra- or inter-molecular interactions of RNA precursors could serve as the substrates of Dicer proteins for endo-siRNA generation in *Drosophila melanogaster*, mice and human. And, a portion of these endo-siRNAs originates from NATs, which is functionally involved in reproduction and neural development^23^,^24^,^25^,^26^,^27^,^28^,^29^,^30^,^37^,^38^. Although the levels, distribution patterns and prevalence of the nat-siRNAs are better characterized in plants, we still hope that the application of NATpipe may extend to the discovery of NATs and nat-siRNAs in animals.

Next, we will make our efforts to improve the functionality of NATpipe, and the presentation of its outputs. For example, the table-based outputs will be converted to graphic ones, taking Fig. 2 as a reference. Besides, the NAT abundances will be considered for expression level-based investigation of the relationships among NATs, nat-siRNAs and degradome signatures from various organs or growth conditions. Summarily, we hope that NATpipe could serve as a useful tool for NAT prediction, nat-siRNA discovery, and related functional studies.

## Additional Information

**How to cite this article**: Yu, D. *et al*. NATpipe: an integrative pipeline for systematical discovery of natural antisense transcripts (NATs) and phase-distributed nat-siRNAs from *de novo* assembled transcriptomes. *Sci. Rep.* **6**, 21666; doi: 10.1038/srep21666 (2016).

## Supplementary Material

Supplementary Information
