# Supplementary material for: NATpipe: an integrative pipeline for systematical discovery of natural antisense transcripts (NATs) and phase-distributed nat-siRNAs from de novo assembled transcriptomes
Source: Sci Rep. 2016 Feb 9;6:21666. doi: 10.1038/srep21666 (PMC4746697; doi:10.1038/srep21666)
Supplement: Supplementary Information [file srep21666-s1.pdf]

NATpipe: an integrative pipeline for systematical discovery of natural antisense transcripts (NATs) and phase-distributed nat-siRNAs from *de novo* assembled transcriptomes

Dongliang Yu[1,&], Yijun Meng[1,2,&, *], Ziwei Zuo[1,2], Jie Xue[1,2], and Huizhong Wang[1,2,*]

1 College of Life and Environmental Sciences, Hangzhou Normal University, Hangzhou 310036, PR China

2 Zhejiang Provincial Key Laboratory for Genetic Improvement and Quality Control of Medicinal Plants, Hangzhou Normal University, Hangzhou 310036, China

[&]These authors contributed equally to this work.

[*]Corresponding authors:

Huizhong Wang

Zhejiang Provincial Key Laboratory for Genetic Improvement and Quality Control of Medicinal Plants, College of Life and Environmental Sciences, Hangzhou Normal University. Xuelin Street 16#, Xiasha, Hangzhou 310036, P. R. China

Tel: +86-571-28865330

E-mail: whz62@163.com

Yijun Meng

Zhejiang Provincial Key Laboratory for Genetic Improvement and Quality Control of Medicinal Plants, College of Life and Environmental Sciences, Hangzhou Normal University. Xuelin Street 16#, Xiasha, Hangzhou 310036, P. R. China

Tel: +86-571-28865198

E-mail: mengyijun@zju.edu.cn

Running Head: Pipeline for NAT and nat-siRNA discovery

Data S1 users' guidelines

Introduction

NATpipe was developed for natural antisense transcript (NAT) prediction and phase-distributed nat-siRNA identification based on transcriptome sequencing data of the organisms without reference genomes.

The input data includes:

- Assembled transcripts (required);
- Small RNA high-throughput sequencing data (optional);
- Degradome sequencing data (optional).

The main output results include:

- NAT candidates, including a list of potential *cis*-NATs (if any);
- Clusters of phased nat-siRNAs with degradome sequencing evidences;
- A summarized table showing statistical result of the analysis.

Notes:

(1) The following analyses should be performed by using third-party software tools:

a. Transcript assembly (by using Trinity);
b. BLASTN;
c. Short read (small RNAs and degradome signatures) mapping (by using Bowtie);
d. RNA hybridization (by using RNAplex).

(2) The input format of a small RNA/degradome sequencing data set is required as follows:

“sRNA/degradome sequence normalized count” for each line of the input data set (“TGACTCGAACGAATTAGAGG 76.06” for example). The normalized abundance (in RPM, reads per million) of each sRNA/degradome sequence from a data set is calculated by dividing the raw count of this sRNA/degradome sequence by the total counts of the corresponding sequencing data set, and then multiplied by 10^6.

Then, the input data sets were further formatted by the tool integrated into NATpipe (please refer to **‘Data format conversion’** in the following section **‘Suggestion for data preparation’** for details).

The converted format is introduced as follows:

- For a small RNA

>S_T1_1234_12.5

ATCGATCGATCGATCG

‘S’ claims this is a small RNA sequence;

‘T1’ specifies the sequence is from replicate ‘1’ of sample ‘T’;

‘1234’ is the tag for this small RNA belonging to the sequencing data set ‘T1’;

‘12.5’ is the abundance of this small RNA (in RPM, reads per million).

‘ATCGATCGATCGATCG’ is the sequence of this small RNA.

- For a degradome signature

>D_Y1_4_24.1

ATCGATCGATCGATCG

‘D’ claims this is a degradome signature;

‘Y1’ claims this sequence is from replicate ‘1’ of sample ‘Y’;

‘4’ is the tag for this degradome signature belonging to the sequencing data set ‘Y1’;

’24.1’ is the abundance of this degradome signature (in RPM, reads per million).

‘ATCGATCGATCGATCG’ is the sequence of this degradome signature.

Requirements

(1) Linux/Windows operation systems

- NATpipe, developed by Perl language, is compatible for Linux and Windows operation systems.

(2) BLAST

- NCBI-BLAST is recommended. For large datasets, all-v-all BLAST is suggested to be run by a computer with high-performance.

(3) Perl

- Standard perl
- Bio::perl

(4) Benchmark Dataset

-536,558 transcripts, with the average length of 930 bp (shmrna.fsa).

-23 M small RNA reads (from four tissues of *Dendrobium officinale*, two replicates per tissue) ([GJYH][12].fsa).

-14 M degradome reads (from four tissues of *Dendrobium officinale*, one replicate per tissue) (d[GJYH].fsa).

(5) Time

The Benchmark Dataset took:

- ~24 h to run all-v-all BLAST using 10 CPU, < 5 min to parse the BLAST result.
- about six weeks to perform RNA hybridization analyses using 10 CPU, including long transcripts; ca. 10 min to parse the result (step2).
- ca. 5 min to predict NATs (step3).
- 2 hours to select perfectly anneal regions (step4).
- 7 h to construct nat-siRNA cluster (step5).

-- < 5 min for parse the short reads mapping result (step6).

- 10 min to get summary information (step7).
- < 5 min to get the statistics report.

Download and installation of third-party software tools

Some third-party software tools were integrated into our pipeline, including NCBI-BLAST [1], RNAplex (ViennaRNA package) [2] and Bowtie [3].

These software tools could be achieved from:

1. NCBI-BLAST 2.2.26

   ftp://ftp.ncbi.nlm.nih.gov/blast/executables/release/

2. Vienna package 2

   http://www.tbi.univie.ac.at/RNA/#download

3. BOWTIE 1.0.0

   Bowtie-bio.sourceforge.net/index.shtml

Please refer to their own users' manual when installing and running the above tools. Notably, this pipeline is developed based on the specified versions of these tools, but the other versions of these tools might be acceptable if their output results are organized with the same manner.

References:

[1]. **Altschul SF, Madden TL, Schaffer AA, Zhang J, Zhang Z, Miller W, Lipman DJ.** 1997. Gapped BLAST and PSI-BLAST: a new generation of protein database search programs. Nucleic Acids Res **25:** 3389-3402.

[2]. **Hofacker IL.** 2003. Vienna RNA secondary structure server. Nucleic Acids Res **31:** 3429-3431.

[3]. **Langmead B, Trapnell C, Pop M, Salzberg SL.** 2009. Ultrafast and memory-efficient alignment of short DNA sequences to the human genome. Genome biology **10:** R25.

Overview of the analytical steps

This is an overview of each analytical step when running NATpipe. All of the steps can be started by using the script "**NATpipe.pl**".

For the users do not have small RNA and degradome sequencing data, only step 1 to 3 are executable.

1. Parsing the result from BLASTN.
2. Parsing the result from RNAplex.
3. Comparing the result of RNAplex with that of BLASTN, and reporting the NAT candidates.
4. Searching for NAT pairs with perfectly annealed regions.
5. Mapping small RNA and degradome sequences to the NATs identified in Step 4.
6. Searching for phased siRNA clusters within the perfectly annealed regions based on small RNA mapping results.
7. Searching for evidences to support the siRNAs processed from the NATs based on degradome mapping results.
8. Generating a summarized table and a detailed report for the identified NATs and nat-siRNAs.

Steps in details

The following scripts can be run independently or started by "**NATpipe.pl**".

Step1. Parsing the result of BLASTN

Usage: blastparser.pl <seqfile> <blastout> < prefix>

Input:

<seqfile>: all transcripts, multiple fasta format;

<blastout>: the output of NCBI-BLASTn, tabular (-m 8);

<prefix>: a tag used to specify the output files.

Output:

prefix_hc.out: alignment of preliminary 'high coverage' type NATs;

prefix_100nt.out: alignment of preliminary '100-nt' type NATs;

prefix_10K.out: transcripts longer than 10 Kb;

prefix.allpairs: a list of all the preliminary NATs (without long transcripts).

Note:

1) Fields of the output files (*.out) is the same as NCBI-BLASTN (-m 8).
2) Pairs containing transcripts longer than 10 Kb are not suggested (but allowed) to be selected for further analyses, as they are time-consuming during hybridization.
3) ‘prefix.allpairs’ is a list of transcript pairs prepared for RNA hybridization analyses.

Example:

>blastparser.pl shmrna.fsa shmrna.blastn BN

*The output files including: BN_hc.out, BN_100nt.out, BN_10K.out and BN.allpairs.

Step2. Parsing the result of RNAplex

Usage: hybrid_parser.pl < RNAplex result > <bubble percent> < output file>

<RNAplex result>: result from RNA hybridization analyses (by RNAplex).

<bubble percent>: [0-1], the percentage of the largest bubbles in annealed regions of both query and reference; 0.1 is suggested.

<output file>: details of checked hybridization result.

The <output file> is tabular formatted, with a total of 13 columns:

seqA: identifier of target sequence;

seqB: identifier of query sequence;

seqA_s/e: start/end site of the hybrid region on seqA;

seqB_s/e: start/end site of the hybrid region on seqB;

energy: minimum free energy;

largest_bubble_seqA/B: length (bp) of the largest bubble in seqA/B within the hybrid region;

hybrid_seqA/B: details of RNA-RNA hybridization in dot-bracket format;

state: ‘OK’ indicates the largest bubbles in both strands are smaller than the threshold.

Example:

>hybrid_parser.pl hcRNAplex.out 0.1 hcRNAplex.parse

Step3. Comparing results between BLASTN and RNAplex, and reporting the NAT candidates

Usage: NATpred.pl -n <parsed blastn> -m <parsed hybridization> -s <mRNA file> -c <overlap percent> -p <prefix>

-n: parsed blastn result for preliminary hc/100nt NATs;

-m: parsed hybridization result for preliminary hc/100nt NATs;

-s: nucleotide sequences of transcripts (fasta);

-c: overlap of blastn and hybridization defined hybrid regions (0-1,default 0.8);

-p: prefix of output files.

The <output file> is tabular formatted, with a total of 19 columns, briefly,

seqA/seqB: transcripts identifier;

BNseqA_s/e: start/end site of seqA from BLASTN alignment;

BNseqB_s/e: start/end site of seqB from BLASTN alignment;

HDseqA_s/e: start/end site of seqA from RNA hybridization;

HDseqB_s/e: start/end site of seqB from RNA hybridization;

OverlapseqA_s/e: start/end site of overlapped region on seqA;

OverlapseqB_s/e: start/end site of overlapped region on seqB;

Energy: minimum free energy of RNA hybridization;

cov_seqA/B: (length of overlapped region) / (length of hybrid region) of seqA/B;

hybrid_seqA/B: details of RNA-RNA hybridization in dot-bracket format.

Example:

>NATpred.pl -n BN_hc.out -m hcRNAplex.parse -s shmrna.fsa –p HC

The output files including: HC.natlist and HC.cisnatlist. HC.natlist includes all the predicted NATs derived from the input files and HC.cisnatlist lists the assumed *cis*-NATs.

Step4. Identification of the perfectly annealed regions of NAT pairs potentially encoding phased siRNAs

Usage : segselection.pl <input file> <prefix> <mini length> <transcripts>

<input file>: details of predicted NATs

<mini length>: minimum length required for searching siRNA cluster encoded regions; 80 is suggested.

<prefix>: to specify the output files

<transcripts>: file including all transcripts.

The output file prefix.PAR is tabular formatted, with a total of 18 columns. Property of the first **17** columns is the same as the output of step3. The **18th** column '**Perfect_annealed_regions'** indicates the regions with the potential to encode nat-siRNA clusters.

The output file prefix.fsa contains the sequences of transcripts included in prefix.PAR. If small RNA and degradome sequencing have been performed, these reads are then mapped to transcripts in prefix.fsa **before starting step5** to identify the evidenced nat-siRNAs.

Example:

>Segselection.pl HC.natlist 80 HC shmrna.fsa

Step5. Identification of the phased siRNA clusters within the perfectly annealed regions

Usage: get_SRcluster.pl <sRNA mapping> <selected segments><phase number><output>

<sRNA mapping>: result from short reads mapping(.bwt);

<selected segments>: details of perfectly annealed regions;

< phase number>: minimum phase number required for a siRNA cluster;

<output>: details of the nat-siRNA clusters.

The output file contains 9 columns, including:

Cluster_ID: This is an internal ID of the phase-distributed small RNA (sRNA) cluster within the specific annealed region of an NAT pair;

Strand(-/+): transcripts;

HD_region(+/-): hybrid regions of transcripts;

Start_phase(+): the site of start phase in strand (+), (5’->3’);

Start_phase(-): the site of the start phase in strand (-), (3’->5’);

No._of_phases: Number of phase-distributed sRNA duplexes;

siRNA_cluster: coordinate of identified phase-distributed sRNAs.

Note:

In the column ‘siRNA_cluster’ of output file, phase-coordinates are split by a comma. ‘q’ and ‘r’ indicate the strand, e.g.,

212_235_q,188_211_q,164_187_q,96_119_r(140_163_q)

‘**212_235_q**’ means at least one small RNA read is mapped onto the region 212-235 of strand (-), but no small RNA has been mapped onto the strand (+) within that duplex.

‘**96_119_r(140_163_q)’** indicates that at least one small RNA is mapped onto each strand within this duplex, with the region 96-119 of strand (+) and 140-163 of strand (-).

Example:

>get_SRcluster.pl GJYH.bwt HC.PAR 4 HC_GJYH.cluster

Step6. Parsing the result of short reads mapping

Usage: perl ShortReadsMappingParse.pl <input> <output>

<input>: result of short reads mapping (*.bwt);

<output>: short reads mapped to transcripts.

This script is used to extract the mapped short reads (both small RNAs and degradome reads). The output file is two columns formatted, splitting by a tab. The first column is the transcript identifier and the second column contains the identifier of all the mapped short reads.

Example:

>ShortReadsMappingParse.pl GJYH.bwt GJYH.parse

Step7. Generating a summarized table and a detailed report for the identified NATs and nat-siRNAs

Usage : getsummary.pl -s <sRNA mapping> -d <degradome mapping> -c <clusters>

-p <prefix> -t <tissues>

-s: parsed result of sRNA mapping;

-d: parsed result of degradome reads mapping;

-c: predicted clusters;

-p: prefix to specify the output files;

-t: tissues ('ABC' represents tissues 'A', 'B' and 'C').

Note:

The input files of -s and -d are generated in step6, -c is generated in step5. The tissues users wanted to check are entered using parameter -t, with each tissue representing by one character. The summary report, prefix.summary includes all the information of degradome sequencing data-supported, phase-distributed small RNAs identified within the perfectly annealed region of NAT pairs. It’s OK if you are concerning only some of the tissues, just specify by the parameter ‘-t’.

Example: >Getsummary.pl –s GJYH.parse –d dGJYH.parse –c HC_GJYH.cluster –p HC –t GJYH

Suggestions for data preparation

1. Data format conversion

Unified identifier for a short read (either a small RNA or a degradome sequence) is required before running NATpipe. A Perl script (**ShortReadsFormat.pl**) is provided in this package to help users formatting the sRNA and degradome sequencing data files.

Usage: ShortReadsFormat.pl <input> <output> <sequence type [S|D]> <sample>

<input>: two-column format file(sequence and FPKM);

<output>: fasta format file;

<sequence types>: S/D; S:small RNA; D:degradome reads;

<sample>: one letter and one digit, e.g.,'T1'.

Examples:

>ShortReadsFormat.pl test.txt test.fsa S J1

Input file (test.txt):

GACACGACTCTCGGCAA 109499.69

TCGGACCAGGCTTCATTCCCC 7029.53

AAGTGGAACATAAAAGAGCCT 5696.48

...

Output file (test.fsa)

>S_J1_1_109499.69

GACACGACTCTCGGCAA

>S_J1_2_7029.53

TCGGACCAGGCTTCATTCCCC

>S_J1_3_5696.48

AAGTGGAACATAAAAGAGCCT

...

2. BLAST

All-v-all BLAST is required for selection the preliminary NATs. After installation of NCBI-BLAST, the transcript file should be formatted firstly, e.g.,

>formatdb –i shmrna.fsa –p F

And then, all-v-all BLAST is performed with the result print out in tabular format, e.g.,

>blastall –i shmrna.fsa –d shmrna.fsa –p blastn –F F –m 8 –o shmrna.blastn

3. RNAplex

In this pipeline, RNAplex from ViennaRNA package is used to perform RNA hybridization analyses. After correctly installation, RNAplex is started like:

>RNAplex -q qry.fa -t ref.fa -l 5000 >outfile

Given a large amount of transcript pairs in the queue, another script (MultipleRNAplex.pl) is provided to perform batched analyses.

Usage: MultipleRNAplex.pl <seqdb> <pairs> <output>

<seqdb>: all the transcripts selected for RNA hybridization analyses;

<pairs>: all the preliminary NATs;

<output>: the hybridization of listed pairs;

Example:

>MultipleRNAplex.pl transcripts.fsa BN.allpairs allRNAplex.out

Note:

The file <pairs> has been generated in step1 (prefix.allpairs). If the transcript pairs are of great number, we suggest users to split the list and start the tasks in parallel. Before starting MultipleRNAplex.pl, please make sure the path of RNAplex is correct.

4. BOWTIE

Simple use of bowtie is required for the pipeline when mapping short reads (small RNAs & degradome reads) to the transcripts, mainly, two steps, take the benchmark dataset for example:

1) index building

>bowtie-build shmrna.fsa SH

2) Short reads mapping

>bowtie -f -a -v 0 --al G1_aligned --un G1_unaligned --norc SH.ebwt G1.fas G1.bwt

Others

The followed two scripts are used to abstract the small RNA or degradome evidence of nat-siRNAs. This function has been included in step 7.

1. GetDetail_sRNA.pl

Usage: GetDetail_sRNA.pl <sRNAparse> <cluster file> <prefix>

<sRNAparse>: parsed result of small RNA mapping;

<cluster file>: the details of nat-siRNA clusters;

<prefix>: used to specify the output file.

2. GetDetail_degradome.pl

Usage: GetDetail_degradome.pl <degparse> <clust file> <prefix>

<degparse>: parsed result of degradome reads mapping;

<clust file>: the details of nat-siRNA clusters;

<prefix>: used to specify the output file.

3. Pipestat.pl

This script is used to get the statistics data of the whole pipeline. A list of files are required, including the NAT list, the PAR list and the details of short reads mapping. These parameters are passed by file "**FilesToStatistic**". Make sure the paths of files (in quotes) are correct before starting Pipestat.pl.

Usage: Pipestat.pl <input file> <output file>

The <input file> is exemplified by "FilesToStatistic". Notably, only in quotes contents are allowed to be modified.

Table S1 Example of output result showing degradome-seq data-supported, phase-distributed nat-siRNAs identified within the perfectly annealed region of an NAT pair in *Dendrobium officinale*.

Cluster ID[1]	Strand (+)[2]	Strand (-)[2]	Annealed region_(+)[3]	Annealed region_(-)[3]	Start phase_(+)[4]	Start phase_(-)[4]	NO. of phases[5]
**116**	**comp175659_c0_seq1**	**comp168422_c0_seq11**	**1904_2956**	**1053_1**	**2429_2449**	**510_530**	**20**

Phase No.[6]	sRNA[7]								Degradome[8]							
	G (root)[9]		J (stem)[9]		Y (leaf)[9]		H (flower)[9]		G (root)[9]		J (stem)[9]		Y (leaf)[9]		H (flower)[9]	
	Strand (+)[10]	Strand (-)[10]	Strand (+)[10]	Strand (-)[10]	Strand (+)[10]	Strand (-)[10]	Strand (+)[10]	Strand (-)[10]	Strand (+)[10]	Strand (-)[10]	Strand (+)[10]	Strand (-)[10]	Strand (+)[10]	Strand (-)[10]	Strand (+)[10]	Strand (-)[10]
**3' extension of strand (-)**	--	--	--	--	--	--	--	--	--	--	--	--	--	--	--	--
**1**	--	--	--	--	--	S_Y2_1504573_0.14_510_530[11]	--	--	D_G1_697549_0.16_2429_2460[11]	--	--	--	--	--	--	--
**2**	--	--	--	--	--	S_Y2_2010950_0.14_489_509	--	--	D_G1_515507_0.16_2450_2481	--	D_J1_503899_0.25_2450_2481	--	D_Y1_841592_0.18_2450_2491	--	--	--
**3**	S_G2_59143_1.27_2471_2491	--	S_J1_76892_0.84_2471_2491/S_J2_174553_0.42_2471_2491	--	S_Y1_1066397_0.17_2471_2491/S_Y2_227212_0.28_2471_2491	--	S_H1_1202484_0.18_2471_2491	S_H1_1689767_0.18_468_488	D_G1_678_131.63_2471_2502	--	D_J1_1592_20.85_2471_2502	--	D_Y1_414_107.79_2471_2512	--	D_H1_507_126.35_2471_2498	--
**4**	S_G2_83282_0.76_2492_2512	--	S_J1_795689_0.09_2492_2512/S_J2_1008896_0.1_2492_2512	--	S_Y1_76529_0.83_2492_2512/S_Y2_301882_0.28_2492_2512	--	--	--	D_G1_21287_3.96_2492_2523	--	D_J1_610656_0.87_2492_2523	--	D_Y1_1593162_0.79_2492_2533	--	D_H1_1281880_0.4_2492_2519	--
**5**	S_G2_130407_0.51_2513_2533	--	S_J1_776635_0.09_2513_2533/S_J2_983827_0.1_2513_2533	--	S_Y1_206864_0.33_2513_2533	--	--	--	D_G1_601685_1.15_2513_2544	--	D_J1_3964886_0.05_2513_2544	--	D_Y1_1902612_0.35_2513_2554	--	--	--
**6**	--	--	--	--	S_Y1_857189_0.17_2534_2554	--	S_H2_825971_0.18_2534_2554	--	D_G1_89492_1.15_2534_2565	--	D_J1_411124_0.2_2534_2565	--	--	--	--	--

**7**	S_G1_780139_0.2_2555_2575/S_G2_24427_4.07_2555_2575	--	S_J1_488623_0.19_2555_2575	--	S_Y2_52641_1.12_2555_2575	--	S_H1_984162_0.18_2555_2575	--	D_G1_245532_1.48_2555_2586	--	D_J1_278319_0.61_2555_2586	--	D_Y1_70413_1.05_2555_2596	--	D_H1_203607_0.4_2555_2582	--
**8**	S_G2_331253_0.25_2576_2596	--	--	--	--	--	--	--	D_G1_1288405_0.16_2576_2607	--	--	--	--	--	--	--
**9**	S_G2_184676_0.51_2597_2617	--	--	--	S_Y2_2139767_0.14_2597_2617	S_Y2_904644_0.14_342_362	S_H2_136460_0.72_2597_2617	--	D_G1_195882_0.99_2597_2628	--	D_J1_1263850_0.2_2597_2628	--	D_Y1_801187_0.18_2597_2638	--	D_H1_2627290_0.13_2597_2624	--
**10**	--	--	S_J2_3666341_0.1_2618_2638	--	--	--	--	--	D_G1_451970_0.66_2618_2649	--	D_J1_1018703_0.31_2618_2649	--	D_Y1_427278_0.35_2618_2659	--	D_H1_862409_0.53_2618_2645	--
**11**	S_G2_86041_0.76_2639_2659	--	--	--	S_Y1_1840890_0.17_2639_2659/S_Y2_837164_0.14_2639_2659	--	S_H2_161851_0.54_2639_2659	--	D_G1_238552_0.99_2639_2670	--	D_J1_1958479_0.15_2639_2670	--	D_Y1_279066_0.44_2639_2680	--	D_H1_206981_0.27_2639_2666	--
**12**	S_G2_99811_0.76_2660_2680	--	--	--	--	--	--	--	D_G1_373771_2.14_2660_2691	--	D_J1_2451624_0.46_2660_2691	--	D_Y1_79567_2.19_2660_2701	--	D_H1_202193_3.32_2660_2687	--
**13**	S_G2_191337_0.51_2681_2701	--	--	--	--	--	--	--	--	--	D_J1_2741400_0.05_2681_2712	--	--	--	--	--
**14**	S_G2_291418_0.25_2702_2722	--	--	--	S_Y1_1517911_0.17_2702_2722	--	--	--	D_G1_299285_0.66_2702_2733	--	D_J1_1049358_0.15_2702_2733	--	D_Y1_2283620_0.09_2702_2743	--	--	--
**15**	S_G2_51073_1.53_2723_2743	--	S_J1_1155778_0.09_2723_2743	--	S_Y1_1069874_0.33_2723_2743	--	S_H1_1206344_0.18_2723_2743/S_H2_995778_0.36_2723_2743	--	D_G1_1243072_0.33_2723_2754	--	--	--	--	--	D_H1_947138_0.13_2723_2750	--
**16**	S_G1_1392517_0.2_2744_2764/S_G2_30392_2.8_2744_2764	--	--	--	S_Y1_59096_1.17_2744_2764/S_Y2_250077_0.28_2744_2764	--	--	--	D_G1_457632_0.16_2744_2775	--	--	--	--	--	--	--
**17**	S_G2_106501_0.76_2765_2785	--	--	--	S_Y1_1402167_0.17_2765_2785/S_	--	--	--	D_G1_453110_0.33_2765_2796	--	--	--	--	--	D_H1_3326709_0.13_2765_2792	--

					Y2_204860_0.28_2765_2785											
**18**	--	--	--	--	S_Y1_1835591_0.17_2786_2806	--	--	--	D_G1_1126136_0.66_2786_2817	--	--	--	--	--	--	--
**19**	S_G2_151911_0.51_2807_2827	--	--	--	S_Y1_772020_0.17_2807_2827/S_Y2_192787_0.28_2807_2827	S_Y2_218212_0.28_132_152	S_H1_230460_0.37_2807_2827	--	D_G1_43042_0.49_2807_2838	--	--	--	--	--	--	--
**20**	S_G1_719263_0.2_2828_2848/S_G2_14488_6.87_2828_2848	--	S_J1_45174_1.03_2828_2848/S_J2_1204936_0.1_2828_2848	--	S_Y1_98398_0.67_2828_2848/S_Y2_85472_0.56_2828_2848	--	--	--	D_G1_1189963_0.16_2828_2859	--	D_J1_3855995_0.05_2828_2859	--	D_Y1_2037504_0.09_2828_2869	--	--	--
**3' extension of strand (+)**	--	--	--	--	--	--	--	--	D_G1_895150_0.16_2849_2880	--	--	--	D_Y1_114340_0.35_2849_2890	--	--	--

[1]This is an internal ID of the phase-distributed small RNA (sRNA) cluster within the specific annealed region of an NAT pair.

[2]"Strand (+)" and "Strand (-)" represent the two transcripts (comp175659_c0_seq1 and comp168422_c0_seq11) forming the NAT pair respectively.

[3]"Annealed region_(+)" and "Annealed region_(-)" provide the positional information of the RNAplex-predicted annealed region on "Strand (+)" and "Strand (-)" respectively.

[4]"Start phase_(+)" and "Start phase_(-)" provide the positional information of the 5' first phases of the sRNA clusters on "Strand (+)" and "Strand (-)" respectively.

[5]Number of phase-distributed sRNA duplexes.

[6]Serial numbers of the continuously phase-distributed sRNA duplexes. Since a total of 20 phased duplexes were discovered as showing in "NO. of phases", serial numbers from "1" to "20" were needed for coding the 20 phases. Notably, "3' extension of strand (-)" and "3' extension of strand (+)" indicate the degradome signatures detected at one nucleotide after the 3' ends of the phased sRNA clusters on "Strand (-)" and "Strand (+)" respectively.

[7]The columns below "sRNA" provide the detailed information of phase-distributed sRNAs identified from sRNA HTS data sets.

[8]The columns below "Degradome" provide the detailed information of the degradome signatures supporting the processing of the phased sRNAs from the NAT pair.

[9]Both sRNA and degradome sequencing data sets were classified into different groups according to the different attributes of the biological samples prepared for sequencing library construction. In this case study, four organs [root (G), stem (J), leaf (Y) and flower (H)] of *Dendrobium officinale* were collected for sequencing. For sRNA sequencing, there are two biological replicates.

[10]The columns "Strand (+)" and "Strand (-)" provide the detailed information of phase-distributed sRNAs or degradome signatures mapped to the 5' ends of either phase within the annealed region of the two transcripts (comp175659_c0_seq1 and comp168422_c0_seq11) respectively.

[11]"S_Y2_1504573_0.14_510_530" indicates that the phased sRNA, with a given ID 1504573 and expression level at 0.14 RPM (reads per million), was identified from the replication 2 of the leaf sequencing experiment. Since it belongs to the column "Strand (-)", the sRNA was assigned to the region from the 510th nucleotide to the 530th nucleotide of the transcript comp168422_c0_seq11. "D_G1_697549_0.16_2429_2460" indicates that the degradome signature, with a given ID 697549 and expression level at 0.16 RPM, was identified from the replication 1 of the root degradome sequencing experiment. Since it belongs to the column "Strand (+)", the degradome signature was assigned to the region from the 2429th nucleotide to the 2460th nucleotide of the transcript comp175659_c0_seq1.

Note: in roots, the sRNA phases from 11th to 17th (gray background color) were identified as a phased sRNA cluster solely based on the two replicates of root sRNA sequencing experiment; in leaves, the sRNA phases from 1st to 7th and from 14th to 20th (gray background color) were identified as two independent sRNA clusters only based the two replicates of leaf sRNA sequencing experiment.
